# Impact of Laser Therapy on the Quality of Life in Women Living With Polycystic Ovary Syndrome-Associated Hirsutism: An Observational Study

**DOI:** 10.7759/cureus.61125

**Published:** 2024-05-26

**Authors:** Syeda Sakina, Faiza Behram, Sarosh Khan Jadoon, Sarosh Mumtaz, Amna Akbar, Ayesha Ijaz Raja, Sabahat Tasneem

**Affiliations:** 1 Dermatology, Dr. Sakina Clinics, Wah Cantt, PAK; 2 Dermatology, Abbas Institute of Medical Sciences, Muzaffarabad, PAK; 3 General Surgery, Combined Military Hospital, Muzaffarabad, PAK; 4 Dermatology, Combined Military Hospital, Muzaffarabad, PAK; 5 Emergency and Accident, District Headquarter Hospital, Jhelum Valley, Muzaffarabad, PAK; 6 Gynaecology, Combined Military Hospital, Muzaffarabad, PAK; 7 Public Health, Health Services Academy, Islamabad, PAK

**Keywords:** ferriman-gallwey (fg), dermatology life quality index (dlqi), laser-assisted hair removal, hirsutism, polycystic ovary syndrome

## Abstract

Introduction: This study aimed to observe the impact of laser-assisted hair removal (LAHR) on the quality of life in women with polycystic ovary syndrome (PCOS)-associated hirsutism.

Methodology: An observational study was conducted on 172 women living with PCOS at a specialized clinic. The Dermatology Life Quality Index (DLQI) and Ferriman-Gallwey (FG) score were employed to assess the quality of life and severity of hirsutism, respectively. Laser therapy was administered using ruby diode or alexandrite lasers. Follow-up on the DLQI and FG score assessment was done at 12-, 18-, and 24-week post-treatment.

Results: The number of cases that reported stress, anxiety, and depression reduced over time. However, there was no correlation between the patient-reported decrease and DLQI scores. The FG score was significantly related to mental health. The severity of the hirsutism impacted mental health. The regrowth of hair at six months indicated limited long-term efficacy LAHR.

Conclusion: LAHR significantly improves the quality of life in the short term for women living with PCOS. However, the short-term benefit of the therapy indicates a need for research to find new treatment strategies.

## Introduction

Hirsutism is characterized by body hair distributed in a masculine pattern covering about 20% of women’s body surface. Ninety-five percent of hirsutism is caused by polycystic ovary syndrome (PCOS) and idiopathic causes [1]. Hirsutism affects the mental health and quality of life of affected women, whether they have moderate or severe hirsutism. A majority of women believe that hirsutism was a barrier to various daily activities associated with employment, recreation, and interpersonal connections and that the effects were independent of age, marital status, and occupation [2]. Hirsutism also poses a financial burden for the management, so it is not sufficient to view it as a cosmetic problem alone. Patients must have a proper evaluation to identify and treat underlying aetiologies and related sequels. The underlying etiology, hormonal drive, and local tissue sensitivity to androgens are among the patient characteristics that determine the efficacy of this therapy, which varies widely [3].

Unwanted hair growth has been historically managed by different methods including waxing, shaving, and epilation, but these are not long-term solutions. Light-based technology, photo epilation, and laser hair removal have been clinically implemented for the past decade and are more effective than conventional methods [4]. Oral contraceptives are first-line treatment for mild to moderate hirsutism, and anti-androgen can be added to enhance effectiveness. Due to teratogenicity, anti-androgens cannot be used as first-line treatment. Physical hair removal techniques are advised by current guidelines [1]. When hirsutism and acne coexist, retinoids for acne and antiandrogen receptor blockers, eflornithine, cyproterone acetate, oral contraceptives for both, and photo epilation for hair removal can be used [5]. A longer course of medication is necessary to improve hirsutism as hair follicles have a lengthy life cycle. Methods of hair removal and changes in lifestyle could enhance the therapeutic response [6].

Currently recognized as a viable therapy option for hirsutism is laser-assisted hair removal (LAHR). Both the physical symptoms and distress associated with hirsutism can be improved by LAHR [7]. Because selective photo thermolysis is safe and effective when used by specialists with the necessary training and experience, it has completely changed the field of laser hair removal. The commercial laser devices for hair removal that have been examined the most are long-pulsed ruby (694 nm), long-pulsed alexandrite (755 nm), diode (800-980 nm), and long-pulsed Nd:YAG (1064 as the wavelength) [8]. In this study, we aimed to observe the impact of LAHR on the quality of life in women with PCOS-associated hirsutism using a questionnaire comprising the Dermatology Life Quality Index (DLQI).

## Materials and methods

The study enrolled 172 female participants presenting with facial hirsutism at the specialized laser clinic at Abbas Institute of Medical Sciences (AIMS), a tertiary care hospital in Muzaffarabad Azad Kashmir, from April 2023 to January 2024. Inclusion criteria comprised women aged 15-70 years with dark brown or black hair and fair skin (Fitzpatrick skin types I, II, and III). The participants were excluded if they had any contraindications to laser therapy or were pregnant. Prior to treatment initiation, informed consent was obtained from all participants. All patients were reported to the hospital OPD, and forms were filled by the participants. AIMS Institutional Review Board issued approval (reference number: 2635).

Ferriman-Galway (FG) score was used to determine hirsutism. The hair growth was examined over nine areas, including the upper lip, chin, chest, upper and lower back, upper and lower abdomen, arms, and thighs. Hair growth in each area is measured on a scale from 0 to 4 as no hair (0), sparse hair (1), moderate hair (2), heavy hair (3), and very heavy hair (4). A score that ranges from 0 to 7 indicates normal or mild hair growth, scores 8-15 exhibit moderate, while scores above 15 indicate severe hirsutism. Women with FG score of at least 8 or a single region score of 4 were considered high cases in the present study. Laser therapy was administered using various systems, including long-pulsed ruby diode or long-pulsed alexandrite lasers, either individually or in combination, as per the manufacturer's recommended parameters. Treatments were aimed at producing per follicular erythematic without blistering, and no local anesthetics were required during the procedures.

The participants were asked to complete a comprehensive questionnaire comprising the unmodified DLQI to assess the baseline quality of life and treatment efficacy side effects, hair regrowth patterns, and the duration between laser sessions. The questionnaire contains 10 questions, each with a score of 0 to 3. The value of DLQI ranges from 0 to 30 and can be divided into the following categories: 0-1 means no impact on patients' life, 2-5 means mild impact, 6-10 means a moderate impact, 11-20 means an adverse impact, and more than 21 means a severe impact on the patients' quality of life.

A subgroup of participants completed the modified DLQI immediately before the treatment and at varying intervals post-treatment (12, 18, and 24 weeks). The DLQI scores were analyzed to evaluate changes in the quality of life over time with statistical analysis performed using paired data and the Mann-Whitney test.

## Results

**Figure 1:**
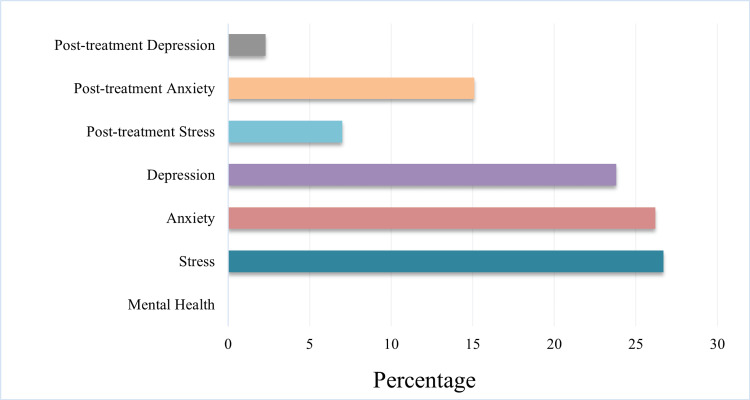
DLQI score pattern Percentage of participants showing specific attributes following treatment. DLQI: Dermatology Life Quality Index

Of the 172 participants enrolled in the study, the majority exhibited a significant improvement (p < 0.05 ) in DLQI scores after 24 weeks of laser therapy (Figure 1).

The median age at which the patients reported was 40 years. At the start of treatment, 46 (26.7%) of the 172 patients were suffering from stress and 41 (23.8%) suffered depression. Stress and depression decreased to 12 (7.0%) and four (2.3%), respectively, after the treatment at 24 weeks (Table 1).

**Table 1:** Association of the Dermatology Life Quality Index (DLQI) score and stress (quality of life versus stress)

Dermatology Life Quality Index		N (%)	Reported stress (n)	p-value
1DLQI at the start of treatment (effect on patients’ life)	Mild effect	4 (2.3 %)	2	0.442
	Moderate effect	27 (15.7%)	6	
	Adverse effect	107 (62.5%)	28	
	Very large effect	33 (19.2%)	10	
DLQI at 12 weeks (effect on patients’ life)	Mild effect	31 (18.5%)	2	0.481
	Moderate effect	68 (40.7%)	3	
	Adverse effect	64 (38.3%)	7	
	Very large effect	4 (2.3%)	0	
DLQI at 18 weeks (effect on patients’ life)	Mild effect	56 (33.3%)	4	0.409
	Moderate effect	87 (51.7%)	4	
	Adverse effect	25 (14.8%)	3	
DLQI at 24 weeks (effect on patients’ life)	Mild effect	58 (34.5%)	5	0.796
	Moderate effect	85 (50.5%)	6	
	Adverse effect	23 (13.8%)	1	

Missing/non-followed-up values were reported to be 1 at the start of treatment, 5 at 12 weeks, 4 at 18 weeks, and 6 at 24 weeks. These responders did not fill the form at the said time frame and thus were not included in the results.

Looking at the DLQI score at different times during the treatment, the score varied across the treatment duration. At the start of the treatment, hirsutism was reported to adversely affect 107 (62.5%) patients’ routine life, while 64 (38.3%), 25 (14.8%), and 23 (13.8%) patients reported adverse effects of the physical appearance due to PCOS-induced hirsutism at 12, 18, and 24 weeks respectively (Table 2). The quality of life reported by the patients at the start of the treatment and during the treatment had no association with stress reported in patients. It probably can be attributed to other symptoms of PCOS, but we are not studying them.

**Table 2:** Association of the Dermatology Life Quality Index (DLQI) score and anxiety (quality of life vs. anxiety)

Dermatology Life Quality Index		N (%)	Reported anxiety (n)	p-value
DLQI at the start of treatment (effect on patients’ life)	Mild effect	4 (2.3%)	3	0.275
	Moderate effect	27 (15.7%)	7	
	Adverse effect	107 (62.5%)	28	
	Very large effect	33 (19.2%)	7	
DLQI at 12 weeks (effect on patients’ life)	Mild effect	31 (18.5%)	3	0.700
	Moderate effect	68 (40.7%)	12	
	Adverse effect	64 (38.3%)	9	
	Very large effect	4 (2.3%)	1	
DLQI at 18 weeks (effect on patients’ life)	Mild effect	56 (33.3%)	10	0.644
	Moderate effect	87 (22.0%)	11	
	Adverse effect	25 (14.8%)	3	
DLQI at 24 weeks (effect on patients’ life)	Mild effect	58 (34.9%)	12	0.188
	Moderate effect	85 (51.2%)	13	
	Adverse effect	23 (13.8%)	1	

Although the number of cases reporting the adverse effect due to hirsutism on patients’ daily lives decreased with treatment, there was no association between the patient-reported DLQI score and pre- or post-treatment anxiety. In both pre- and post-treatment surveys, the maximum number of patients who reported anxiety were those who reported having an adverse effect on daily life. The reported number of pre-treatment events of anxiety was 28 at the start of the treatment (Table 3).

**Table 3:** Association of the Dermatology Life Quality Index (DLQI) score and depression (quality of life vs. depression)

Dermatology Life Quality Index		N (%)	Reported depression (n)	p-value
DLQI at the start of treatment (effect on patients’ life)	Mild effect	4 (2.3%)	1	0.822
	Moderate effect	27 (15.7%)	5	
	Adverse effect	107 (62.2%)	25	
	Very large effect	33 (19.2%)	10	
DLQI at 12 weeks (effect on patients’ life)	Mild effect	31 (18.4%)	0	0.447
	Moderate effect	68 (40.4%)	1	
	Adverse effect	64 (38.0%)	3	
	Very large effect	4 (2.3%)	0	
DLQI at 18 weeks (effect on patients’ life)	Mild effect	56 (33.3%	1	0.831
	Moderate effect	87 (51.7%)	2	
	Adverse effect	25 (14.8%)	1	
DLQI at 24 weeks (effect on patients’ life)	Mild effect	58 (34.5%)	1	0.566
	Moderate effect	85 (50.5%)	3	
	Adverse effect	23 (13.6%)	0	

The trend with depression was the same as anxiety, there was no association between the patient-reported DLQI score and pre- or post-treatment depression. In both pre- and post-treatment surveys, most patients who reported depression were those who reported having an adverse effect on daily life. The reported number of post-treatment events of depression was three and one, respectively, at 12 and 18 weeks (Table 4).

**Table 4:** Association of the Ferriman-Gallwey (FG) score and stress (hirsutism vs. stress)

Ferriman-Gallwey (FG) score		N (%)	Reported stress (n)	p-value
FG-score at start of treatment	Normal/mild hirsutism	14 (8.1%)	6	0.010
	Moderate hirsutism	56 (32.5%)	7	
	Severe hirsutism	102 (59.3%)	33	
FG-score at 12 weeks	Normal/mild hirsutism	23 (14.1%)	1	0.870
	Moderate hirsutism	93 (57.0%)	5	
	Severe hirsutism	47 (28.8%)	6	
FG-score at 18 weeks	Normal/mild hirsutism	58 (34.7%)	1	0.235
	Moderate hirsutism	92 (55.0%)	9	
	Severe hirsutism	17 (10.1%)	2	
FG-score at 24 weeks	Normal/mild hirsutism	96 (40.3%)	3	0.043
	Moderate hirsutism	74 (43.2%)	9	
	Severe hirsutism	1 (0.5%)	0	

The FG score measures the severity of hirsutism. According to the results of the present study, the level of stress in patients was associated with the severity of hirsutism, and the association was observed to be significant at the start of the treatment (p = 0.010) and at the 24th week of the treatment (p = 0.043) (Table 5). The number of patients with severe hirsutism decreased and those with mild increased, showing a positive impact of the laser treatment.

**Table 5:** Association of the Ferriman-Gallwey (FG) score and anxiety (hirsutism vs. anxiety)

Ferriman-Gallwey (FG) score		N (%)	Reported anxiety (n)	p-value
FG-score at the start of treatment	Normal/mild hirsutism	14 (8.1%)	3	0.684
	Moderate hirsutism	56 (32.6%)	16	
	Severe hirsutism	102 (59.3%)	26	
FG-score at 12 weeks	Normal/mild hirsutism	23 (14.1%)	2	0.169
	Moderate hirsutism	93 (57.0%)	12	
	Severe hirsutism	47 (28.8%)	11	
FG-score at 18 weeks	Normal/mild hirsutism	58 (34.7%)	2	0.002
	Moderate hirsutism	92 (55.0%)	22	
	Severe hirsutism	17 (10.1%)	1	
FG-score at 24 weeks	Normal/mild hirsutism	96 (56.1%)	8	0.014
	Moderate hirsutism	74 (43.2%)	18	
	Severe hirsutism	1 (0.6%)	0	

In the postoperative survey, when the level of anxiety was determined and the relation with the FG score was assessed, the scores at 18 weeks (p = 0.002) and 24 weeks (p = 0.014) were significantly associated with anxiety. The number of hirsute women facing anxiety was 45 at the start of the treatment. Out of the 45 women who reported anxiety at the start of treatment, 26 reported anxiety post-treatment too. Similarly, the number of cases reporting anxiety was reduced to almost half at all points in time during the treatment. From the results in Table 6, it is evident that the severity of hirsutism shifted from severe to mild during the treatment.

**Table 6:** Association of the Ferriman-Gallwey (FG) score and depression (hirsutism vs. depression)

Ferriman-Gallwey (FG) score		N (%)	Reported depression (n)	p-value
FG-score at the start of treatment	Normal/mild hirsutism	14 (8.1%)	4	0.245
	Moderate hirsutism	56 (32.6%)	8	
	Severe hirsutism	102 (59.3%)	29	
FG-score at 12 weeks	Normal/mild hirsutism	23 (14.1%)	0	0.214
	Moderate hirsutism	93 (57.0%)	4	
	Severe hirsutism	47 (28.8%)	0	
FG-score at 18 weeks	Normal/mild hirsutism	58 (34.7%)	0	0.020
	Moderate hirsutism	92 (55.5%)	2	
	Severe hirsutism	17 (10.1%)	2	
FG-score at 24 weeks	Normal/mild hirsutism	96 (56.1%)	0	<0.001
	Moderate hirsutism	74 (43.2%)	3	
	Severe hirsutism	1 (0.5%)	1	

The patients reported depression at the start of the treatment and among the 107 patients who had severe hirsutism before treatment, 29 reported having depression. The FG score significantly reduced over the course of treatment. The most marked effect was at the 18th (p = 0.020) and 24th weeks (p < 0.001)

Following laser therapy, the DLQI scores demonstrated a notable reduction at 12 to 24 weeks post-treatment. Hair regrowth pattern was also assessed with most participants experiencing hair reappearance within one to three months post-treatment. By six months, nearly all participants had unwanted hair back at pretreatment levels, indicating limited long-term efficacy of laser therapy in preventing hair regrowth.

## Discussion

Common signs of hyperandrogenism associated with PCOS include hirsutism, acne, and female pattern hair loss. The first-line treatment for hyperandrogenism symptoms is still combined hormonal contraceptives [9]. For hirsutism to improve, therapy must be administered for a minimum of six to nine months in women with moderate to severe hirsutism, or sudden onset rapidly progressing, or coupled with other abnormalities, such as menstrual dysfunction, obesity, or macroclitoris. Pharmacological therapy or direct hair removal procedures are recommended for women who still have significant hirsutism after cosmetic measures [10]. The management of PCOS causes economic loss, and women may not be able to afford laser hair removal or electrolysis; gym memberships to lose weight; or, in some countries, especially low- and middle-income countries, the birth control pill to fix their cycles [11]. When patients with idiopathic hirsutism seek laser treatment, psychological morbidity is observed to be lower in these cases. The sample DASS 21 scores were 8.21, 4.43, and 4.34 for stress anxiety and depression, respectively (within the normal range) [12].

Excess terminal hair growth over androgen-dependent areas in women is known as hirsutism [13]. The FG score is a gold standard for determining hirsutism and its extent. The present diagnosis of hirsutism does not include the depression associated with it [14]. Estimates of the prevalence of hirsutism in females range from 10% to 20%, resulting in severe psychological harm and social stigma [15]. When hirsute females were surveyed, the Hospital Anxiety and Depression Scale (HADS), DLQI, and FG scale scores were associated with hirsutism [16]. Emotions and symptoms had the highest mean score of the DLQI scale [17]. Alopecia, insulin resistance, hirsutism, and acne are all frequently associated with PCOS. The ratio of facial hair to mustache hair was the most significant indicator of women who are hirsute and have PCOS [18].

Patients receiving laser treatment were observed for six months. A decline in self-reported facial hair severity and reduced time spent on hair removal marked better outcomes in the intervention group. Changes in the mean anxiety scores along with a decrease in the depression score and improved quality of life were also attributed to the intervention group [19]. Intense pulsed light (IPL) can cause an average long-term reduction of hair from 52.7% to 27%. Among laser devices, Alexandrite lasers showed better results than Nd:YAG and diode lasers in terms of hair count reduction [20]. IPL along with radiofrequency (RF) prove more useful for skin phenotypes II and III [21]. For Fitzpatrick skin types III-VI, Alexandrite was more effective than IPL. For the skin phenotype used in this study, laser devices have comparable results.

LAHR can be used safely and efficiently on darker skin types if the laser's parameters, such as wavelength, pulse length, energy fluence, spot size, and repetition rate are carefully chosen. When used appropriately, LAHR can help children and adolescents with hirsutism and hypertrichosis feel less emotionally burdened [22]. When comparing group 3 (laser + OCs) to groups 2 (laser + metformin) and 1 (laser alone), the difference was substantial. The DLQI and HLQI scores were better at three and six months after treatment compared to zero month. Meanwhile, group 2 patients exhibited decline at three months and recovery at six months. Group 1 showed a significant decline only after six months [11].

For the treatment of 40 women with hirsutism (mean DLQI score of 5.55 ± 1.501), 3% showed no effect, 52% showed effect, and 45% showed moderate effect. Symptoms and feelings were the domain with the highest mean DLQI. Furthermore, the LQI and FG scores exhibited a marginally favorable but nonsignificant connection. 

Limitations

The findings of this study corroborate previous research demonstrating the short-term benefits of laser therapy on the quality of life in women with hirsutism. However, the observed plateau treatment efficacy and the limited long-term sustainability of these benefits raise important clinical considerations. Despite the initial reduction in DLQI scores and high patient satisfaction levels, most participants experienced a return to pretreatment levels of hair growth within six months underscoring the need for realistic patient expectations and long-term management strategies. The discrepancy between patient-reported satisfaction and objective measure of treatment efficacy emphasizes the complex interplay between perceived outcome expectations and clinical reality in the context of laser therapy for hirsutism. Meanwhile, laser therapy achieving long-lasting hair reduction warrants further investigation into alternative treatment modalities and combination approaches. Although this is a large study with 172 participants, the numbers amenable to statistics in each specific sub-group type is small, which is a limitation and therefore lacks generalizability.

## Conclusions

Laser therapy represents a valuable tool in the management of hirsutism, and different treatment comparisons offer significant short-term improvements in the quality of life and patient satisfaction. However, the limited long-term efficacy of laser therapy in preventing hair regrowth underscores the need for ongoing research and innovation in this field. Clinicians should counsel patients regarding the realistic expectations and potential limitations of laser therapy while exploring alternative treatment modalities to optimize long-term outcomes and quality of life for women with hirsutism.
